# *LvSlc12A2* Is a Negative Growth Regulator in Whiteleg Shrimp, *Litopenaeus vannamei*

**DOI:** 10.3390/ani15172467

**Published:** 2025-08-22

**Authors:** Panpan Niu, Shanshan Jiang, Mianyu Liu, Siyu Chen, Jie Kong, Sheng Luan, Xianhong Meng, Qun Xing, Qifan Zeng, Kun Luo, Huan Gao

**Affiliations:** 1 State Key Laboratory of Mariculture Biobreeding and Sustainable Goods, Yellow Sea Fisheries Research Institute, Chinese Academy of Fishery Sciences, Qingdao 266071, China; niupanpanvv@163.com (P.N.); 2022213007@stu.njau.edu.cn (M.L.); kongjie@ysfri.ac.cn (J.K.); luansheng@ysfri.ac.cn (S.L.); mengxianhong@ysfri.ac.cn (X.M.); luokun@ysfri.ac.cn (K.L.); 2 Jiangsu Key Laboratory of Marine Biotechnology, Jiangsu Ocean University, Lianyungang 222005, China; 15261305425@163.com (S.J.); csy19551363785@163.com (S.C.); 3 The Jiangsu Provincial Infrastructure for Conservation and Utilization of Agricultural Germplasm, Nanjing 210014, China; 4 BLUP Aquabreed Co., Ltd., Weifang 261312, China; xingqun527@163.com; 5 Ministry of Education Key Laboratory of Marine Genetics and Breeding, College of Marine Science, Ocean University of China, Qingdao 266003, China; zengqifan@ouc.edu.cn

**Keywords:** *Litopenaeus vannamei*, *LvSlc12A2*, RNA interference, overexpression, in situ hybridization

## Abstract

*Litopenaeus vannamei*, commonly known as the Pacific white shrimp, is one of the most economically significant species in global aquaculture, valued for its rapid growth and adaptability. However, the mechanisms regulating its growth, especially under high-density farming and environmental stress, remain poorly understood. Previous study predicted that *LvSlc12A2* was involved in growth regulation. To further reveal the function of this gene in the growth regulation of the whiteleg shrimp, in this study, we explore its function using RT-qPCR, RNA interference, overexpression, and tissue in situ hybridization. RT-qPCR results showed that *LvSlc12A2* was highly expressed in gills (about 62%), followed by the hepatopancreas, with the lowest expression in muscle (0.08%, compared to the gills). Myostatin (*LvMstn*) was mainly expressed in the heart, and molt-inhibiting hormone (*LvMIH*) in the ventral nerve. In situ hybridization of gill tissues using the mRNA of the gene as a probe revealed strong *LvSlc12A2* signals in the gill stratum and epithelial cells. Overexpression of *LvSlc12A2*, significantly decreased the osmotic gene aquaporin (*LvAqp*), while knockdown increased its expression. Additionally, levels of growth-related inhibitory genes *LvMstn* and *LvMIH* increased significantly after *LvSlc12A2* overexpression and were downregulated after its knockdown, suggesting *LvSlc12A2* negatively regulates growth, possibly in synergy with *LvMstn* and *LvMIH*. These findings indicate *LvSlc12A2* influences growth both by negative regulation and by modulating osmotic balance in gill tissues.

## Simple Summary

The *LvSlc12A2* gene plays an important role in osmotic regulation, cell volume regulation, and neural signal transduction, though its role in the growth and development of crustaceans remains unclear. In a previous transcriptome analysis, we found that *LvSlc12A2* was significantly differentially expressed in the fast- and slow-growing families of whiteleg shrimp, *Litopenaeus vannamei*. Therefore, we postulate that this gene may also be closely related to the regulation of growth and development in *L. vannamei*. Our results showed that *LvSlc12A2* can synergize with *LvMstn* and *LvMIH* genes in function, and participate in the growth regulation of *L. vannamei* through negative regulatory mechanisms. *LvSlc12A2* antagonized the *LvAqp* gene and regulated the growth of *L. vannamei* by participating in osmotic pressure regulation in gill tissues. Our study, showing that *LvSlc12A2* is a negative regulator of *L. vannamei* growth, offers a novel genetic target for aquaculture breeding strategies.

## 1. Introduction

Solute carrier (SLC) transporters are a large family of more than 400 membrane-bound proteins, the second largest group of membrane transporters after G protein-coupled receptors. They facilitate the transport of various substrates across biological membranes and play an important role in physiological processes from cellular uptake of nutrients to uptake of drugs and other exogenous substances [1]. The SLC family is divided into 65 subfamilies that mediate the exchange of substances such as ions, nutrients, signaling molecules, and drugs across biological membranes. SLC12 is a complete small family of membrane proteins, consisting of *SLC12A1*-*SLC12A9* genes that encode an electrically neutral, cation-conjugated chloride transporter [2,3]. To date, all SLC12 family members that have been functionally characterized have been shown to be electrically neutral, cation-chloride cotransporters [4]. Nine SLC12 family members are known to be in vertebrates [5], including two NKCCs [6,7] (NKCC1 and NKCC2), a Na^+^-Cl^−^ cotransporter (NCC), four K-Cls (KCC1, KCC2, KCC3, and KCC4), and two other proteins with unclear functions (CIP and CCC9). Studies have shown that the Na^+^-K^+^-2Cl^−^ cotransporter, NKCC1 (encoded by *LvSlc12A2*) is capable of transmembrane ion transport at a ratio of 1Na:1K:2Cl [8,9]. It plays a role in osmotic regulation under different salinity stresses, maintains cell ion homeostasis and regulates cell volume, and thus maintains cellular osmolality [5,6,7,10]. NKCC1 depletion decreases the intracellular Na^+^ concentration and cell volume (size) and quality, and stimulates cell proliferation [4]. NKCC1 transports Na, K, and Cl ions between epithelial and non-epithelial cells, thereby regulating cell proliferation, differentiation, and metastasis [11]. The protein is found in the stomach, esophagus, colorectal, liver, pancreas, lungs, and other tissues, and is abnormally expressed in many tumors. In addition, the SLC12 sequence has been identified in many lower species, including crustaceans, insects, worms, plants, fungi, and some bacteria. In recent years, significant progress has been made in the study of Solute Carrier (SLC) family genes in *Litopenaeus vannamei*, revealing their crucial roles in various key physiological processes. In the context of immune defense, studies have found that *LvSlc15A4* participates in the innate immune response by activating the NF-κB signaling pathway [12], while *LvSlc5* has been confirmed to be associated with resistance to White Spot Syndrome Virus (WSSV) infection [13]. Furthermore, regarding environmental stress responses, the functions of several SLC members have also been elucidated; for instance, both *LvSlc26A6* [14] and *LvSlc12A2* [15] have been shown to be involved in the response to nitrite stress. However, although the *LvSlc12A2* gene has been cloned and its function in stress response reported, its potential role in the regulation of growth in *L. vannamei* and the underlying molecular mechanisms remain unknown, presenting an important scientific question for further investigation.

*L. vannamei* occupies an important position in China’s aquaculture, and is widely cultivated because of its strong adaptability and tolerance to extensive culture. It is the shrimp species with the highest aquaculture yield in the world [16,17]. With the intensified competition in the *L. vannamei* farming industry, the cultivation of fast-growing new varieties has always been an important target for industrialization. The growth rate of *L. vannamei* is affected by a variety of internal and external factors, such as water-salt balance, hormone regulation, etc., but gene regulation is a crucial part of it. In a previous study (unpublished data), we established fast-growing and slow-growing families of *L. vannamei* and conducted transcriptome analysis with the aim to find genes related to growth regulation. We found that the *LvSlc12A2* gene was significantly differentially expressed in the fast- and slow-growing families. This finding prompted further investigation into the role and mechanism of this gene in regulating shrimp growth, with the goal of accelerating the breeding of improved fast-growing varieties. To achieve this, we used a combination of quantitative PCR (qPCR), RNA interference (RNAi), gene overexpression, and tissue in situ hybridization to explore the expression patterns of *LvSlc12A2* in different tissues, and its regulatory effects on growth-related inhibitory genes such as *LvMstn* (myostatin) and *LvMIH* (molt-inhibiting hormone). We also assessed the involvement of *LvSlc12A2* in osmoregulatory mechanisms by evaluating its impact on *LvAqp* (aquaporin) expression, providing insights into its potential dual role in growth regulation and osmotic pressure control in *L. vannamei*.

## 2. Materials and Methods

### 2.1. Source of Materials

The shrimps used in this study were from BLUP Aquabreed Co., Ltd., Weifang, China. Shrimps were reared in tanks measuring 60 cm (L) × 50 cm (W) × 40 cm (H). Each tank was stocked with 30 shrimp. All individuals had a good growth status and consistent specifications, with an average body length of 7 ± 0.2 cm and an average body weight of 3 ± 0.2 g. Before the experiments, the shrimp were temporarily reared for one week under controlled conditions. The rearing water temperature was maintained at 25.0–28.0 °C, salinity at 24 ± 2 ppt, pH at 8.0 ± 0.2, and dissolved oxygen at 7.0 ± 0.5 mg/L. The shrimp were fed commercial feed produced by Tongwei Group (Chengdu, China), containing approximately 35% crude protein, 8% crude lipid, and 6% ash. Feed was administered twice daily at 9:00 and 16:00, with the feed amounting to 1% of body weight each time. During this period, the water was under 24 h continuous aeration, with regular cleaning of bait and feces. Sex was not considered during the selection process.

### 2.2. RNA Extraction and cDNA Synthesis

Instructions from the relevant kits were used. Total RNA was extracted from 15 to 25 mg of tissue with a UNIQ-10 column Trizol total RNA extraction kit (Sangon, Shanghai, China). The quantity and purity of the extracted RNA were assessed using a NanoDrop spectrophotometer by measuring the absorbance at 260 nm and 280 nm (A260/A280), and the RNA integrity was evaluated by 1% agarose gel electrophoresis. 1 µg of qualified RNA was reverse-transcribed into cDNA using a HiScript^®^ III RT SuperMix for qPCR (+gDNA wiper) (Vazyme, Nanjing, China) kit, and then stored at −80 °C.

### 2.3. Analysis of LvSlc12A2, LvMstn, and LvMIH Expression Characteristics

Nine healthy shrimps were dissected to obtain hemolymph, eyestalk, stomach, heart, gill, hepatopancreas, intestine, muscle, ventral nerve, and ovary tissues. RNA extraction and cDNA synthesis were performed as described in Section 2.2. Specific PCR primers were designed using Primer Premier 5.0 software based on the conserved sequences of the core of the *LvSlc12A2* gene (Table 1), and 18S rRNA was used as the internal reference gene [16], and qRT-PCR was performed according to the ChamQ SYBR qPCR Master Mix (Vazyme, Nanjing, China). The reaction procedure was as follows: 1 cycle of 95 °C for 15 s for pre-denaturation; 40 cycles of 95 °C for 3 s and 60 °C for 30 s. All samples were analyzed in triplicate (n = 3).

### 2.4. LvSlc12A2 Gene Overexpression

Recombinant primers were designed according to the core conserved sequences of the *LvSlc12A2* gene (Table 1), and the overexpression vector was constructed. The constructed vectors were transcribed in vitro according to the Easy Cap T7 Co-transcription Kit with the CAG Trimer Kit (Vazyme, Nanjing, China). Two groups were designed, namely the control group and the overexpression group, with three parallel replicates in each group and 30 animals in each replicate. The control group was injected with only normal saline (saline injection), and the overexpression group was injected with dsRNA of the *LvSlc12A2* gene. The injection volume was prepared as a standard of 4 μg/g normal saline or dsRNA. At 0, 6, 12, 24, 48, and 72 h post-injection, three shrimp were randomly sampled from each tank, and their gill and muscle tissues were, respectively, pooled. Consequently, three biological replicates were obtained for each group at each time point. All quantitative analyses were performed with three technical replicates for each biological sample. Quantitative analysis was performed on key target genes, including two growth inhibition of related genes (*LvMstn* [18] and *LvMIH* [19]) and osmotic pressure regulation (*LvAqp* [20]). The qRT-PCR-related primers are shown in Table 1.

### 2.5. RNA Interference

The *LvSlc12A2* gene interference primers (Table 1) were designed from the online website https://rnaidesigner.thermofisher.com/rnaiexpress/ (accessed on 10 July 2024) (thermofisher.com). RNA interference reagents were synthesized according to the instructions of the T7 RNAi Transcription Kit (Vazyme, Nanjing, China). RNA interference experiments were performed on healthy, vigorous, uniformly sized *L. vannamei*. The experimental method is the same as that described in Section 2.4.

### 2.6. In Situ Hybridization

The recombinant primers were designed according to the core sequence of the *LvSlc12A2* gene. The linearized DNA was amplified with linearized primers and *LvSlc12A2-PSPT18* recombinant plasmid. The primers are shown in Table 1. The DIG RNA labeling kit (SP6/T7) reagent was used for in vitro transcription. After the DIG-labeled probes were synthesized, the integrity of the combined probes was detected by 1% agarose gel electrophoresis. The probe concentration was determined by a nucleic acid analyzer, and the probes were stored at −80 °C for later use.

Gill tissue of *L. vannamei* was fixed in 4% paraformaldehyde for 24 h, and tissues were routinely dehydrated, embedded in paraffin, and sectioned (slice thickness 4 μm). The synthetic mRNA probe of the *LvSlc12A2* gene was used for pre-hybridization and hybridization experiments, and the DAB chromogenic agent was used for color development. After ethanol dehydration and xylene treatment, neutral gum was used to seal the sections, and natural drying was carried out under a microscope, where the hybridization signal was observed and photomicrographed [17].

### 2.7. Data Analysis

All data are presented as mean ± SEM. Datasets were evaluated for the homogeneity of variance (Levene’s test) and for normality (the Shapiro–Wilk test). The qRT-PCR results were statistically analyzed using SPSS 19.0 and the 2^−ΔΔCt^ method with Excel software [21]. The differential expression of the gene among various tissues was analyzed by one-way ANOVA followed by the Least Significant Difference (LSD) post hoc test. Differences between the control group and the interference group were compared using repeated measure two way ANOVA and followed by Bonferroni post hoc tests for multiple comparisons. *p* values less than 0.05 were considered to be significant.

## 3. Results

### 3.1. Expression Characteristics of LvSlc12A2, LvMstn, and LvMIH in Different Tissues of L. vannamei

**Figure 1:**
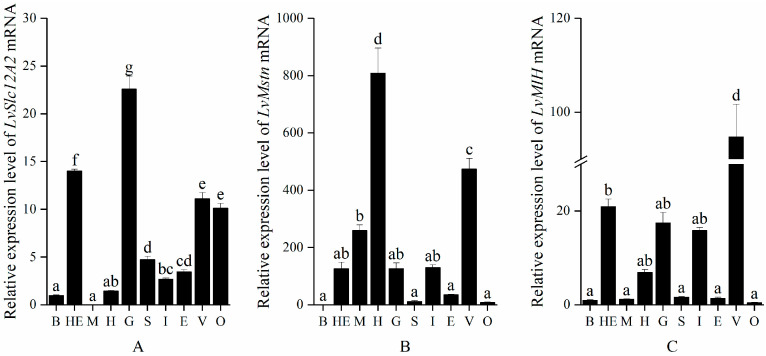
Expression characteristics of genes in different tissues of *L. vannamei*. (**A**) Expression of *LvSlc12A2* in various tissues of *L. vannamei;* (**B**) expression of *LvMstn* in various tissues of *L. vannamei;* (**C**) expression of *LvMIH* in various tissues of *L. vannamei*. Note: B: Blood; HE: hepatopancreas; M: muscle; H: heart; G: gill; S: stomach; I: intestine; E: eyestalk; V: ventral nerve; O: ovary. Data are presented as mean ± SEM (n = 3). Different lowercase letters above the bars indicate significant differences among treatments (one-way ANOVA followed by LSD multiple range test, *p* < 0.05).

The tissue distribution of *LvSlc12A2*, *LvMstn*, and *LvMIH* expression was investigated using real-time fluorescence quantification (results are shown in Figure 1). Different tissue-specific expression patterns were seen for all three genes (Figure 1). *LvSlc12A2* (Figure 1A), *LvMstn* (Figure 1B), and *LvMIH* (Figure 1C) were ubiquitously expressed across the ten examined tissues in *L. vannamei*, but distinct expression patterns were seen with significant tissue specificity. *LvSlc12A2* was highly expressed in the gills (*p* < 0.05), followed by the hepatopancreas. It comprised about 62% of the expression in the gills, and the lowest expression level was in muscle (only 0.08% of that of the gills). *LvMstn* showed the highest expression in the heart, and the expression in the muscle was about 32% of that of the heart. The lowest expression was in the blood. *LvMIH* was highly expressed in nervous tissue, followed by the hepatopancreas, intestine, gills, and heart, with the lowest expression in the blood.

### 3.2. Effects of LvSlc12A2 Overexpression on the LvAqp, LvMstn, and LvMIH Genes

**Figure 2:**
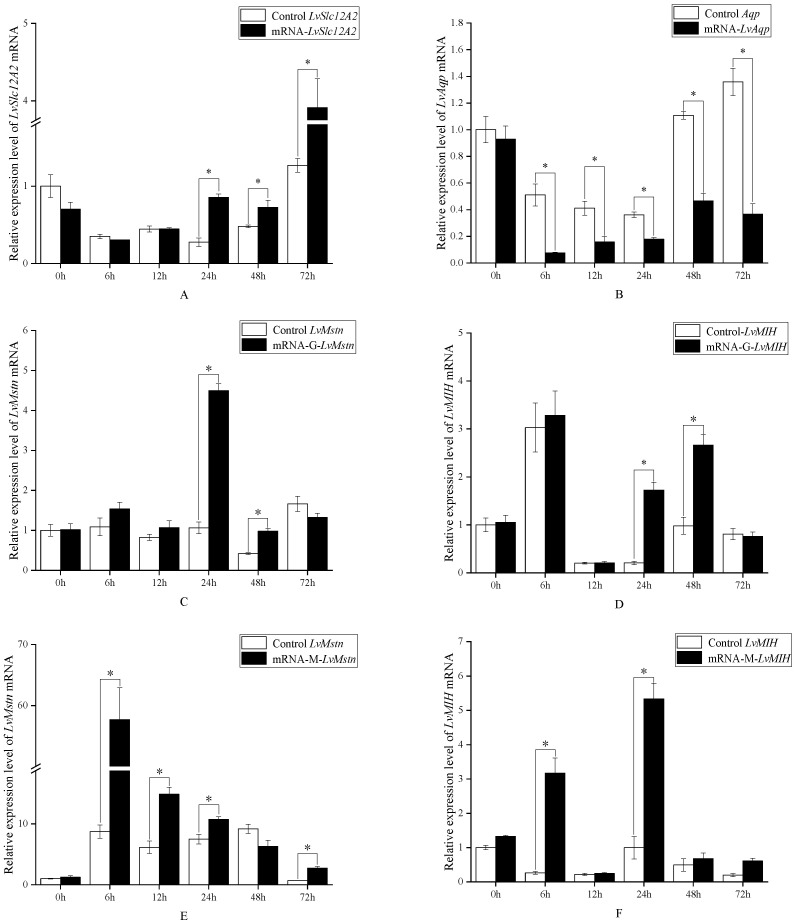
Relative expression levels of each gene at different time points after overexpression of *LvSlc12A2*. (**A**) Expression levels of *LvSlc12A2* in gills after overexpression treatment; (**B**) expression levels of *LvAqp* in gills after overexpression treatment; (**C**) expression levels of *LvMstn* in gills after overexpression treatment; (**D**) expression levels of *LvMIH* in gills after overexpression treatment; (**E**) *LvMstn* expression levels in muscle after overexpression treatment; (**F**) *LvMIH* expression levels in muscle after overexpression treatment. Data are presented as mean ± SD (n = 3). The asterisk (*) indicates a significant difference between the two groups (repeated measure two-way ANOVA followed by Bonferroni post hoc tests for multiple comparisons, *p* < 0.05).

Repeated measure two-way ANOVA confirmed that *LvSlc12A2* overexpression, time, and their interaction significantly affected the expression of target genes (Figure 2). In gill tissue, we first confirmed the successful overexpression of *LvSlc12A2* at 24, 48, and 72 h, with the highest levels observed at 72 h (Figure 2A). This overexpression led to a significant downregulation of *LvAqp* (Figure 2B) at 6, 12, 24, 48, 72 h and a significant upregulation of both *LvMstn* (Figure 2C) and *LvMIH* (Figure 2D) at 24 and 48 h. In muscle tissue, the expression of *LvMstn* and *LvMIH* was also significantly altered. *LvMstn* levels were elevated across multiple time points (6, 12, 24, and 72 h), peaking at a 6.6-fold increase at 6 h (Figure 2E). *LvMIH* expression was also robustly induced, rising by approximately 12-fold and 7.5-fold at 6 and 24 h, respectively (Figure 2F).

### 3.3. Effects of Knockdown LvSlc12A2 Genes on LvAqp, LvMstn, and LvMIH Genes

**Figure 3:**
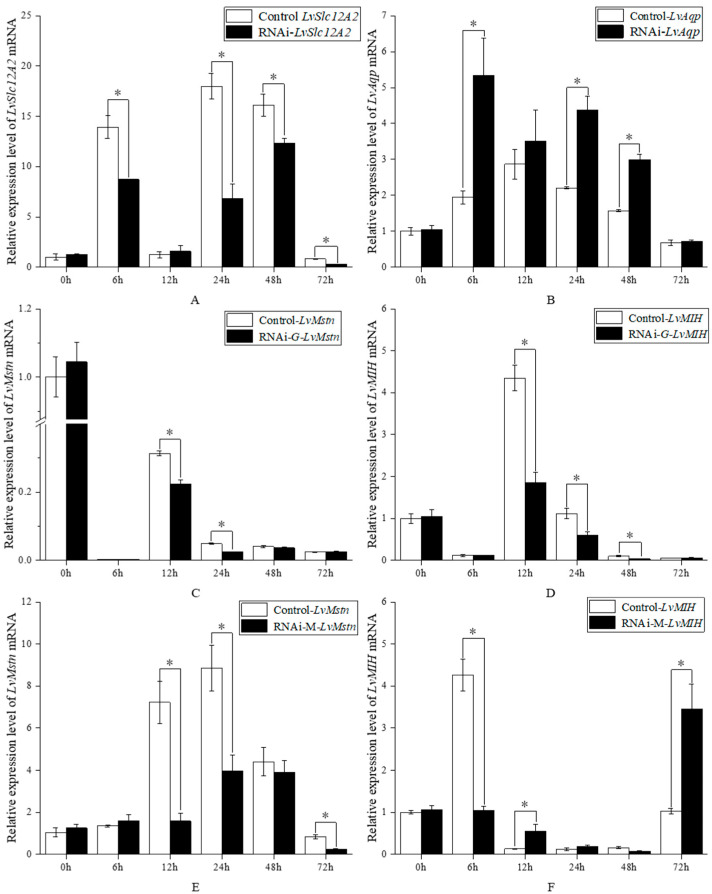
Relative expression levels of each gene at different time points after knockdown *LvSlc12A2*. Notes: (**A**) expression levels of *LvSlc12A2* in gills after RNAi treatment; (**B**) *LvAqp* expression levels in gills after RNAi treatment; (**C**) expression levels of *LvMstn* in gill tissues after RNAi treatment; (**D**) expression levels of *LvMIH* in gills after RNAi treatment; (**E**) *LvMstn* expression levels in muscle after RNAi treatment; (**F**) *LvMIH* expression levels in muscle after RNAi treatment. Data are presented as mean ± SD (n = 3). The asterisk (*) indicates a significant difference between the two groups (repeated measure two-way ANOVA followed by Bonferroni post hoc tests for multiple comparisons, *p* < 0.05).

Silencing of the *LvSlc12A2* gene via RNA interference significantly altered the expression of several related genes in both gill and muscle tissues (Figure 3). These effects were significantly influenced by treatment, time, and their interaction (repeated measure two-way ANOVA, *p* < 0.05). In the gills, *LvSlc12A2* expression was effectively suppressed at all tested time points (6, 24, 48, and 72 h), with a maximum knockdown efficiency of 70.2% observed at 24 h (Figure 3A). This knockdown resulted in the significant upregulation of *LvAqp* at 6, 24, and 48 h (Figure 3B), and the significant downregulation of *LvMstn* (at 12 and 24 h) and *LvMIH* (at 12, 24 and 48 h) (Figure 3C,D). In muscle tissue, *LvMstn* levels were consistently repressed at 12, 24, and 72 h following *LvSlc12A2* knockdown (Figure 3E). In contrast, *LvMIH* exhibited a biphasic response, showing significant downregulation at 6 h, followed by significant upregulation at 12 and 72 h (Figure 3F).

### 3.4. Localization of LvSlc12A2 mRNA in Gill Tissue

**Figure 4:**
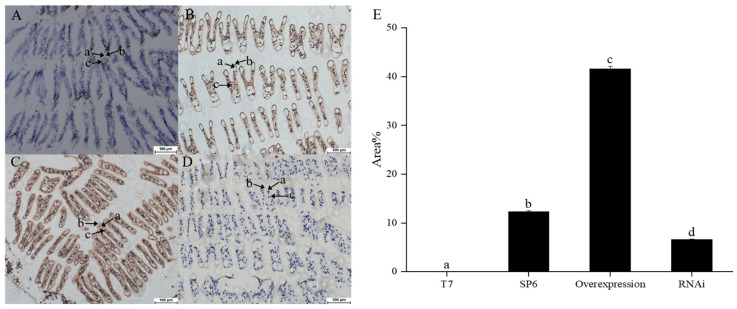
Spatial distribution of *LvSlc12A2* mRNA in gill tissue of *L. vannamei.* (**A**) Localization results for the sense probe in gill tissue. (**B**) Localization results for the antisense probe in gill tissue of the control group. (**C**) The mRNA-*LvSlc12A2* groups of the ISH assay. (**D**) The RNAi-*LvSlc12A2* groups of the ISH assay. (**E**) Semi-quantitative ISH analysis showing the expression pattern of *LvSlc12A2* in gill tissue. Note—a, epithelial cells; b, stratum corneum; c, nucleus. Data are presented as mean ± SEM (n = 3). Different lowercase letters above the bars indicate significant differences among treatments (one-way ANOVA followed by LSD multiple range test, *p* < 0.05).

The spatial distribution of the *LvSlc12A2* gene in the gills of *L. vannamei* were analyzed using the mRNA of *LvSlc12A2* as a probe. The results, shown in Figure 4, demonstrate that the hybridization signal of the *LvSlc12A2* gene mainly appears in the gill epithelium and cuticle cells in gill tissue, and no hybridization signal appears in the nucleus.

This study also detected the expression distribution with a semi-quantitative analysis of *LvSlc12A2* in gill tissues of each treatment group (Figure 4A–E). The results show that the positive signal in the mRNA-*LvSlc12A2* group (Figure 4C) was stronger, compared to the blank control group (Figure 4B). In addition, the positive signal in the RNAi-*LvSlc12A2* group (Figure 4E) was weaker, compared to the blank control (Figure 4B).

## 4. Discussion

### 4.1. Distribution Characteristics and Function of LvSlc12A2

To explore the biological function of *LvSlc12A2*, qRT-PCR was used to analyze the expression of *LvSlc12A2* in different tissues of *L. vannamei*. The results show that the *LvSlc12A2* gene was expressed in all tissues, with the highest expression in gill tissue, followed by the hepatopancreas, and the lowest expression was in muscle. To further explore the spatial expression distribution of *LvSlc12A2* in gill tissue, we used in situ hybridization to locate the expression of the mRNA of *LvSlc12A2* in gill tissue, and found that the hybridization signals were mainly concentrated in the gill epithelium and cuticle cells. This is consistent with the high expression level of *LvSlc12A2* that was found in gill tissues of *Penaeus japonicus* and *tilapia* [15,22,23,24], supporting the idea that *LvSlc12A2* may be involved in osmotic regulation in the gills. Notably, its expression in muscle was the lowest among all tested tissues. We speculate that this low expression may indicate that *LvSlc12A2* plays only a minor role in muscle physiology, possibly reflecting the lower involvement of muscle tissue in osmotic balance, while still participating indirectly in growth and metabolic regulation through systemic signaling. The gills are a multifunctional organ that play a key role in respiration, acid-base balance regulation, ion balance maintenance, nitrogen excretion, and osmoregulation [25]. The gill epithelium contains many cell types, such as granulosa cells, nephrogenic cells, and columnar cells, etc., for regulating and maintaining the osmotic balance between the external water environment and the hemolymph. The cells are known to perform functions in respiration, excretion, osmotic pressure regulation, and even disease prevention [26,27]. The coordinated transport of SlC12A2 located on the plasma membrane of the basal side of these cells also plays an important role [28,29,30,31]. Besides epithelial cells, *LvSlc12A2* can also be expressed in non-epithelial cells such as heart and skeletal muscle neurons [32]. The expression of *LvSlc12A2* has also been reported in the choroid cereus parietal membrane, neurons, oligodendrocytes, and dorsal root ganglion neurons in the nervous system [33]. Therefore, because of its involvement with perception of normal sensations, we speculate that *LvSlc12A2* may also be associated with hormonal regulation.

### 4.2. Regulatory Links Between LvSlc12A2 and the Expression of LvMstn and LvMIH

Our finding that *LvSlc12A2* regulates the expression of both *LvMstn* and *LvMIH* provides a novel mechanistic link between cellular ion homeostasis and the complex regulation of shrimp growth. This connection is particularly significant because *LvMstn* and *LvMIH* are the primary regulators of the two distinct modes of crustacean growth: continuousand discontinuous. The broad, non–tissue-specific expression of *LvMstn* [18,34,35,36] and the wide distribution of *LvMIH* beyond traditional neurosecretory sites [19] align with their central roles in systemic growth control—a process our findings now implicate *LvSlc12A2* in modulating.

A plausible mechanism for this modulation is grounded in the established functions of *LvSlc12A2*. Both muscle development and the substantial tissue expansion during molting demand precise regulation of cell volume. As a key controller of ion flux and cell volume [2,37,38], *LvSlc12A2* is well positioned to meet these demands. Moreover, its capacity to influence key metabolic pathways—such as by limiting mTORC1 activity [39]—suggests it could directly shape the cellular states required for both sustained tissue growth and successful molting. We therefore propose that *LvSlc12A2* functions not merely as a housekeeping ion transporter, but as a strategic regulatory node, integrating fundamental cellular physiology with high-level developmental signals from *LvMstn* and *LvMIH* to ensure balanced and efficient growth in shrimp.

### 4.3. LvSlc12A2 Biological Function in the Growth and Development of L. vannamei

This study reveals for the first time that the ion transporter *LvSlc12A2* plays a dual role in *L. vannamei*, acting as a crucial node that links osmoregulation with growth control. The *LvAqp* (aquaporin) family comprises small and very hydrophobic intrinsic membrane proteins with functions such as water transport, cell osmotic regulation, osmotic stress response, and selective ion osmosis [40]. Our results demonstrate an inverse relationship between *LvSlc12A2* and the aquaporin gene *LvAqp*, which is consistent with the function of SLC12 family members in managing ion gradients that influence cellular water balance and trigger compensatory expression of aquaporins under osmotic stress. Most studies have found that changes in osmotic pressure can regulate gene expression in tissues [41]. For example, NKCC in *Eriocheir sinensis* responds rapidly to external osmotic changes [42]. Interfering with *LvAqp1* expression reduces water transport in the gills, resulting in an osmotic difference between the inside and outside of cells; while promoting NKCC upregulates the gill Na^+^/K^+^-*ATP* gene, facilitating excess ion excretion [43]. Therefore, altering *LvSlc12A2* expression may change osmotic pressure and affect *LvAqp* expression, suggesting its role in osmoregulation. Concurrently, we found a positive correlation between the expression of *LvSlc12A2* and the growth-inhibiting genes *LvMstn* and *LvMIH*. RNAi-mediated suppression of *LvSlc12A2* reduced their expression, whereas its overexpression significantly increased their levels in both gill and muscle tissues. Collectively, these findings suggest that *LvSlc12A2* is not just a housekeeping transporter but a key signal integrator, capable of translating physiological stress signals, such as osmotic shifts, into commands that modulate the pace of growth and molting.

A key observation from our time-course analysis is the tissue-specific dynamics of this regulation, with muscle tissue exhibiting a more pronounced response to *LvSlc12A2* modulation compared to the gills. For instance, both overexpression and knockdown elicited a much larger fold-change in *LvMstn* expression in muscle tissue (Figure 2C,E vs. Figure 3C,E), following overexpression, while *LvMstn* expression increased significantly in both tissues at 6 h, the change in muscle tissue was 6.6-fold, relative to the control, and in the gills, it was only 1.4-fold, highlighting muscle as a more sensitive target of this pathway. A similar trend was observed for *LvMIH* (Figure 2D,F vs. Figure 3D,F). The most striking tissue-specific phenomenon was the late-stage response of *LvMIH* in muscle. While overexpression led to a sustained high level of *LvMIH*, the knockdown condition produced an unexpected, significant upregulation at 72 h (Figure 2F). We hypothesize that Prolonged ionic imbalance may trigger a neuroendocrine feedback loop that activates *LvMIH* expression to restore homeostatic control over the vital molting cycle, a complex reaction not observed in the more rapidly compensating gill tissue. Another possibility is that environmental fluctuations induced stress responses, as suggested by Figure 3, which shows gene expression variability at different time points in both experimental and control groups. Such variation may reflect stress-induced self-protective or tissue-repair signaling in shrimp.

Our findings also contribute to the ongoing debate regarding the precise role of myostatin (*Mstn*) in crustacean growth. *LvMstn*, a myostatin gene, regulates muscle growth mainly via the TGF-β/Smad and PI3K-AKT-mTOR pathways, acting as a negative regulator of muscle differentiation, growth, and protein synthesis, and has been shown to inhibit growth in most crustaceans [44,45]. Some studies have also shown that *LvMstn* has a positive regulatory effect on muscle growth during the molting cycle (e.g., in *L. vannamei*), and the growth rate can be slowed down after inhibiting *LvMstn* expression [18,46,47]. Nevertheless, some research also suggests that *LvMstn* negatively regulates the growth of *L. vannamei* [48], and it still remains unclear whether *LvMstn* is a positive or negative regulator of crustacean muscle growth [49]. In *Penaeus monodon*, the RNAi-mediated knockdown of *PmMstn* resulted in significantly slower growth relative to the control group, suggesting that *PmMstn* may play a positive regulatory role in growth [34]. In *Fenneropenaeus merguiensis*, elevated *FmMstn* expression was seen in smaller individuals of the same age group, indicating its possible negative regulatory role in muscle development [50]. In *Fenneropenaeus chinensis*, about 20–30% of the individuals in the dsRNA-injected group molted after injection at about 12 h, indicating that down-regulated of *FcMstn* can increase the molt for growth [51]. *LvMIH* is a molting-inhibiting hormone belonging to the crustacean hyperglycemic hormone (CHH) family of neuropeptides, which negatively regulate the molting growth of crustaceans by inhibiting ecdysone synthesis in the Y-organs [52]. In this study, we showed that after interfering or overexpressing *LvSlc12A2*, *LvMstn* and *LvMIH* showed similar expression patterns, which is reasonable since these genes are related to the molting cycle [35,53]. Our findings reveal that the analogous expression patterns of *LvMstn* and *LvMIH* imply a shared functionality in growth regulation. We have also demonstrated that *LvMstn*, pivotal for muscle growth and development, likely acts as a negative regulator of growth in shrimp, mirroring its conserved function in vertebrates. We speculate that the up-regulation or down-regulation of *LvSlc12A2* may inhibit or activate the pathways where *LvMstn* and *LvMIH* genes are located, in turn, changing their levels of expression. Our results show that the three factors likely play a synergistic role in the growth and development of *L. vannamei*, jointly regulating its growth.

While our molecular data provide compelling evidence for this regulatory network, a limitation of this study is that we did not directly measure the resulting growth phenotypes. Based on our findings, we hypothesize that elevated basal expression of *LvSlc12A2* would correlate with slower growth, possibly indicating that there was an abnormal or stressed physiological state in the shrimp. This hypothesis is consistent with observations in other biological systems where low level of expression of *LvSlc12A2* homologs (NKCC1) is characteristic of normal, mature physiology [37]. Therefore, future research should not only validate the phenotype link but also identify the upstream factors that cause variations in *LvSlc12A2* expression. Potential mechanisms warranting investigation include epigenetic regulation, such as DNA methylation, which has been shown to control SLC12A expression [34,40]. Furthermore, given the established roles of the SLC12A family in neurodevelopment and injury response [54], it is plausible that the shrimp’s neurological health could also contribute to *LvSlc12A2* dysregulation and consequently affect growth. Elucidating these upstream pathways holds significant potential for developing novel biomarkers for selecting robust and fast-growing shrimp in aquaculture.

## 5. Conclusions

In this study, we detected the expression of *LvSlc12A2*, *LvMstn*, and *LvMIH* genes in various tissues by real-time fluorescence quantification, and measured the expression of *LvAqp*, *LvMstn*, and *LvMIH* in gill and muscle tissues at different time points after overexpression and interference. We found that *LvSlc12A2* was highly expressed in gill tissues, *LvMstn* was expressed in the heart, and *LvMIH* was expressed in the ventral nerve. Our in situ hybridization results showed that the gene was mainly expressed in gill epithelial cells and in the stratum corneum. The relative expression of *LvAqp* decreased significantly and *LvMstn* and *LvMIH* expression increased significantly after the overexpression of the *LvSlc12A2* gene. The converse was also true in that after *LvSlc12A2* interference, the relative expression of *LvAqp* increased significantly and the relative expressions of *LvMstn* and *LvMIH* decreased significantly. Although the specific regulatory mechanisms remain unknown, our results indicate that *LvSlc12A2* negatively regulates the growth of *L. vannamei*.

## Figures and Tables

**Table 1:** Sequence of oligonucleotide used in this study.

Primer Name	Sequences	Genbank Accession No.	Length (bp)	Efficiency (%)	Purpose
*18S*-F	TATACGCTAGTGGAGCTGGAA	EU920969	147	92.1	Internal reference
*18S*-R	GGGGAGGTAGTGACGAAAAAT				
*LvMstn*-F	GGGACTTCATTGTTGCTC	JQ045427.1	120	97.62	Real-time fluorescent
*LvMstn*-R	CGCTGGTGCTATTCATCT				quantitative
*LvMIH*-F	AGCAGTTCAACAGGTGGATCAG	MF358695.1	123	108.82	
*LvMIH*-R	AAGGAGCAGCAGGAGGAGAG				
*LvAqp*-F	GCAGCCATCTTGAAGGGAGTGAC	XM_070140003.1	125	95.96	
*LvAqp*-R	ACGAGGACGAAGGTGATGAGGAG				
*LvSlc12A2*-F	GACTCTCCTGCTGCCTTACATCC	PQ073211	122	91.38	
*LvSlc12A2*-R	GGTTTGCCATGCTTCTCTGTTCC				
S-A1	GATCACTAATACGACTCACTATAGGGCAAACGTAATCAGTCTGCTGGTCAGTT		25		RNA interference
S-A2	AACTGACCAGCAGACTGATTACGTTTGCCCTATAGTGAGTCGTATTAGTGATC				
S-B1	GATCACTAATACGACTCACTATAGGGCTGA CCAGCAGACTGATTACGTTTGTT				
S-B2	AACAAACGTAATCAGTCTGCTGGTCA GCCCTATAGTGAGTCGTATTAGTGATC				
Pspt18-SF	GAGACCGGAATTCGAGCTCGGAACTCTACGGACCTCTTAGAGAACC		1056		Homologous recombination
Pspt18-SR	GAATACAAGCTTGCATGCCTGCCGGTCACACTTAGCCCAATTTG				
SP6-*LvSlc12A2*	ATTTAGGTGACACTATAGAATACAAGCTTGCATGCCTGCCGGTCACACTTAGC		1073		In situ hybridization
T7--*LvSlc12A2*	TAATACGACTCACTATAGGGAGACCGGAATTCGAGCTCGGAACTCTACGGACC		1075		

## Data Availability

The original contributions made in this study are contained within the article; any additional inquiries can be directed to the corresponding authors.
